# Pan-Mitogenomics Approach Discovers Diversity and Dynamism in the Prominent Brown Rot Fungal Pathogens

**DOI:** 10.3389/fmicb.2021.647989

**Published:** 2021-05-12

**Authors:** Gozde Yildiz, Hilal Ozkilinc

**Affiliations:** ^1^School of Graduate Studies, MSc Program in Biomolecular Sciences, Çanakkale Onsekiz Mart University, Çanakkale, Turkey; ^2^Faculty of Arts and Sciences, Department of Molecular Biology and Genetics, Çanakkale Onsekiz Mart University, Çanakkale, Turkey

**Keywords:** pan-mitogenomics, mitogenome, evolution, *Monilinia* species, brown rot

## Abstract

*Monilinia fructicola* and *Monilinia laxa* species are the most destructive and economically devastating fungal plant pathogens causing brown rot disease on stone and pome fruits worldwide. Mitochondrial genomes (mitogenomes) play critical roles influencing the mechanisms and directions of the evolution of fungal pathogens. The pan-mitogenomics approach predicts core and accessory regions of the mitochondrial genomes and explains the gain or loss of variation within and between species. The present study is a fungal pan-mitogenome of *M. fructicola* (*N* = 8) and *M. laxa* (*N* = 8) species. The completely sequenced and annotated mitogenomes showed high variability in size within and between the species. The mitogenomes of *M. laxa* were larger, ranging from 178,351 to 179,780bp, than the mitogenomes of *M. fructicola*, ranging from 158,607 to 167,838bp. However, size variation within the species showed that *M. fructicola* isolates were more variable in the size range than *M. laxa* isolates. All the mitogenomes included conserved mitochondrial genes, as well as variable regions including different mobile introns encoding homing endonucleases or maturase, non-coding introns, and repetitive elements. The linear model analysis supported the hypothesis that the mitogenome size expansion is due to presence of variable (accessory) regions. Gene synteny was mostly conserved among all samples, with the exception for order of the *rps3* in the mitogenome of one isolate. The mitogenomes presented AT richness; however, A/T and G/C skew varied among the mitochondrial genes. The purifying selection was detected in almost all the protein-coding genes (PCGs) between the species. However, *cytochrome b* was the only gene showing a positive selection signal among the total samples. Combined datasets of amino acid sequences of 14 core mitochondrial PCGs and *rps3* obtained from this study together with published mitochondrial genome sequences from some other species from Heliotales were used to infer a maximum likelihood (ML) phylogenetic tree. ML tree indicated that both *Monilinia* species highly diverged from each other as well as some other fungal species from the same order. Mitogenomes harbor much information about the evolution of fungal plant pathogens, which could be useful to predict pathogenic life strategies.

## Introduction

Fungi are one of the most remarkable and diverse kingdom, with approximately 720,256 species compared with other eukaryotic organisms worldwide (Blackwell, 2011; Badotti et al., 2017). Fungal genomics exhibit important data for studies of adaptive behavior and evolutionary research due to their highly dynamic and fast-evolving features. High-throughput sequencing technologies have allowed for sequencing of the tremendous number of fungal nuclear genomes across many species, with a total of 2,599^1^. However, relatively limited data is available for whole mitochondrial genomes. For instance, only 793 mitochondrial genomes have been announced by NCBI Organelle Genome Resources^2^. Mitochondrial genomes present valuable information to explain both adaptative traits and the evolution of pathogens. Fungal mitochondrial genes can be targeted for plant disease management (Medina et al., 2020) and provide specific markers for population studies, as well as species diagnosis (Santamaria et al., 2009). Furthermore, mitogenome data contribute to expand information of fungal phylogenetics (Chen et al., 2019; Nie et al., 2019; Li Q. et al., 2020). Fungal mitochondrial genomes consist of highly conserved proteins and RNA encoding genes related to respiration and translation processes (Aguileta et al., 2014; Franco et al., 2017). Moreover, the presence of mitochondrial-encoded ribosomal protein genes, such as *rps3*, and its homologs (*var1* and *S5*) differs among fungal groups, and these genes may have been transferred to the nuclear genomes in different eukaryotic species (Bullerwell et al., 2000; Smits et al., 2007; Sethuraman et al., 2009; Korovesi et al., 2018; Yildiz and Ozkilinc, 2020). Furthermore, copy number, gene duplications, gain/loss of introns, and transposable and repetitive elements are the main factors causing mitogenome variations (Basse, 2010; Aguileta et al., 2014). Because of these factors, mitogenome sizes may vary within and among fungal taxonomic groups (Burger et al., 2003). Recent studies showed that homing endonucleases, such as GIY-YIG and LAGLIDADG families, play a significant role in shaping fungal genome structure and contribute to variations within and between species (Sandor et al., 2018; Kolesnikova et al., 2019; Yildiz and Ozkilinc, 2020).

Fungal mitogenomes have been evaluated for genomic features (Li Q. et al., 2020), comparative mitogenomics (McCarthy and Fitzpatrick, 2019), and pan-mitogenomics (Brankovics et al., 2018). The pan-genomic approaches, using a comparative genomics-based methodology to identify the core and accessory genomes or genomic regions, were applied on bacterial genomes at first (Tettelin et al., 2008). Core genomes tend to be conserved among strains, such as many housekeeping genes involved in translation, metabolism, and oligopeptide metabolism (McCarthy and Fitzpatrick, 2019). However, the accessory genome includes dispensable, variable, and “unessential regions,” which may not be present in all strains or isolates within a clade (Torres et al., 2020). Thus, genome content can vary in distinct populations of a single fungal species, and the inventory of the variation at the genomic level in different isolates is crucial to characterize the complete set of accessory genes (Stajich, 2017). Two-speed genome evolution is referred to indicate compartmentalization of the fungal genomes as shared and slowly evolving regions as well as variable and fast-evolving regions (Dong et al., 2015; Bertazzoni et al., 2018; Torres et al., 2020). Pangenomics approach has recently resolved different fungal nuclear genomes (Kelly and Ward, 2018; McCarthy and Fitzpatrick, 2019; Badet et al., 2020); however, only few studies focused on mitochondrial genomes. For instance, sequencing of mitogenomes of *Aspergillus* and *Penicillium* species were analyzed by presenting core and accessory genes through comparative mitogenome analyses (Joardar et al., 2012). The mitogenome of phytopathogenic fungus *Fusarium graminearum* was analyzed considering the pan-mitochondrial genomics concept (Brankovics et al., 2018).

*Monilinia* species include phytopathogenic fungi that belong to the *Ascomycota* division. They cause brown rot disease on many stone and pome fruits, which results in severe economic losses around the world. *Monilinia laxa*, *Monilinia fructicola*, and *Monilinia fructigena* are the prevalent pathogenic species of the *Monilinia* genus causing this disease (Holb, 2006, 2008). The complete mitogenome of *M. laxa* was characterized by our previous study for the first time and presented higher content of mobile introns in comparison to some of the other phytopathogenic species from closely related genera (Yildiz and Ozkilinc, 2020). Thus, we expected high mitochondrial diversity within this pathogenic species and its relative, causing the same disease. This study aimed to uncover mitochondrial variations, by pan-mitogenomic approach, of the 16 mitogenomes from the two prominent and most abundant species (*M. fructicola* and *M. laxa*) that are known to cause brown rot disease. Mitogenomes were annotated as well as phylogenetics, and evolutionary selections were evaluated based on the protein-coding regions of the mitogenomes. This provides an essential foundation for future studies on population genetics, taxonomy, and crop protection strategies from the perspective of mitogenomics.

## Materials and Methods

### Fungal Isolates and DNA Extraction

Isolates of *Monilinia* species were selected from the collection of Dr. Ozkilinc. Original isolates were long term stored at −20°C on Whatman filter papers no 1. Isolates were obtained from infected peach fruits from different orchards in six cities of Turkey (Ozkilinc et al., 2020). Sixteen isolates from a large collection of *Monilinia* samples were selected. The list of selected fungal pathogens of *M. fructicola* and *M. laxa* species is represented in Table 1. Selected isolates were grown from their original stored cultures on potato dextrose agar media at 23°C in the dark. Mycelia were transferred to potato dextrose broth and incubated at room temperature on a rotary shaker at 150 rpm for 5–7 days for genomic DNA isolation (Yildiz and Ozkilinc, 2020). Total DNA extractions were carried out using a commercial kit for fungi/yeast genomic DNA isolation (Norgen Cat. 27300, Canada), following the manufacturer’s protocol. Concentration and purity of DNAs were assessed with a spectrophotometer (NanoQuant Infinite M200, Tecan) and a fluorometer (Qubit 3.0, Thermo Fisher Scientific, United States), then the DNAs were sent to an external service for Illumina-based library construction and short-read sequencing (Macrogen Inc., Next-Generation Sequencing Service, Geumcheon-gu, Seoul, South Korea).

**TABLE 1:** The list of selected fungal isolates from *M. fructicola* and *M. laxa* species.

**Isolate Code**	**Species**	**City/Country**	**Host species**	**Orchard number**
B5-A4	*M. fructicola*	Çanakkale/Turkey	Peach	5
T-B1-A5	*M. fructicola*	Izmir/Turkey	Peach	10
Ti-B3-A2	*M. fructicola*	Izmir/Turkey	Peach	3
Ti-B3-A3-2	*M. fructicola*	Izmir/Turkey	Peach	3
Yolkenari-1	*M. fructicola*	Izmir/Turkey	Peach	Greengrocers
SC-B2-A4	*M. fructicola*	Samsun/Turkey	Peach	2
BG-B1-A4	*M. fructicola*	Bursa/Turkey	Peach	1
BG-B1-A17	*M. fructicola*	Bursa/Turkey	Peach	1
2B1-A5	*M. laxa*	Çanakkale/Turkey	Peach	1
T-B1-A4-2	*M. laxa*	Izmir/Turkey	Peach	1
Ni-B3-A2	*M. laxa*	Nigde/Turkey	Peach	3
MM-B2-A2	*M. laxa*	Mersin/Turkey	Peach	2
MM-B4-A4	*M. laxa*	Mersin/Turkey	Peach	4
MT-B1-A3-1	*M. laxa*	Mersin/Turkey	Peach	1
Yildirim-1	*M. laxa*	Bursa/Turkey	Peach	1
Yildirim-2-10th	*M. laxa*	Bursa/Turkey	Peach	–

### Genome Sequences and *de novo* Assembly

The whole-genome sequence libraries of the 16 *Monilinia* spp. isolates were constructed using Illumina platform with TruSeq Nano kit to acquire paired-end 2 × 151 bp with 350-bp insert size, provided by Macrogen Inc., Next-Generation Sequencing Service. Adapters and low-quality reads were removed from raw data by using Trimmomatic 0.36 (Bolger et al., 2014) with the parameters as followed previously (Yildiz and Ozkilinc, 2020). The reads were evaluated to control the quality of sequences by using FastQC (Andrews, 2010), and the quality-checked data were used for further analysis. The mitogenomes were assembled *de novo* and extracted from the complete genome data using GetOrganelle v1.6.2 (Jin et al., 2019) with K-mer value: 105, including the SPAdes v3.6.2 (Bankevich et al., 2012) assembly program. QUAST reports presenting contig sizes, N50, and L50 were checked for the assembled contigs, including possible mitogenomes (Gurevich et al., 2013). The obtained mitogenomes for each isolate were visualized using the Geneious 9.1.8 program (Kearse et al., 2012). Mitogenomes represented by more than one contig were mapped by referring to the other completed mitogenomes using Geneious 9.1.8 (Kearse et al., 2012).

### Mitochondrial Genome Annotations and Gene Orders

Core protein-coding genes (PCGs), ribosomal RNA, transfer RNA, and introns were annotated using the online server MFannot (Beck and Lang, 2010) and Mitos WebServer (Bernt et al., 2013). Mold/Protozoan/Coelenterate mitochondrial genetic code four was used for the annotation of the mitogenomes. Annotation of rRNA and tRNA genes was checked by using RNAweasel (Beck and Lang, 2010) and tRNAscan-SE 2.0 (Lowe and Chan, 2016), respectively. Hypothetical proteins, including ORFs and LAGLIDADG, GIY-YIG homing endonuclease families, were detected in intergenic regions using ORFinder in NCBI and Sequence Manipulation Suite: ORFFinder (Stothard, 2000), as well as the product of possible protein sequences, were checked by smart-blast in NCBI. Possible predicted mitochondrial genes were confirmed by a basic local alignment search tool using a nucleotide blast (BLASTN) in NCBI. The previously annotated mitogenome of *M. laxa* isolate (Ni-B3-A2) (accession number: MN881998) was used as a reference to check the annotations. Annotated gene arrangements were analyzed by using MAUVE 2.3.1 software (Darling et al., 2010). Moreover, duplicate regions in mitogenomes of isolate *M. fructicola* were investigated by using the Geneious 9.1.8 program (Kearse et al., 2012).

### Identification of Repetitive Sequences

The repetitive elements in the mitogenomes were determined by using Tandem Repeats Finder (Benson, 1999). The repetitive sequences and their motifs were compared within and between the species.

### Pan-Mitogenomics Analysis to Predict Conserved and Variable Regions Within Species

The percentages of the conserved and variable regions of the mitogenomes within each species were determined using bioinformatics tools Spine and AGEnt (Ozer et al., 2014). Additionally, R programming language with a deviance function from the stats package (R Core Team, 2013) was applied to interpret the statistical analysis of whether the intragenic intron sizes have contributed to differentiation in mitogenome sizes within the species. Thus, a linear correlation between the mitochondrial genome size (as the dependent variable) and intron length (as the independent variable) was tested based on the null hypothesis of positive correlation expectation.

### Estimation of Codon Usage and Evolutionary Selection Patterns in Mitogenomes of *Monilinia* Species

The non-synonymous (Ka) and synonymous substitution rates (Ks) were calculated for all PCGs by using DnaSP v6.10.01 (Rozas et al., 2017). Since all the coding regions were almost the same within the species, the evolution rate was estimated on the total data set from both species. The strength of selection was inferred by considering that if the calculated ratio is equal to, greater than, or less than 1 indicates neutral evolution, positive (diversifying) selection, or purifying (negative) selection, respectively. Ka/Ks values for all protein-coding regions were visualized with ggplot in R programming language (R Core Team, 2013). The Relative Synonymous Codon Usage (RSCU) was obtained using MEGA 7 software (Kumar et al., 2016) and determined for all coding regions. Furthermore, the nucleotide frequency of occurrence in each protein-coding gene (including the full length of the exons and introns) as well as in genes related to the ribosome (*rnl* and *rns*) was assessed for A/T and G/C asymmetry by using the following formulas:

AT⁢skew=(A-T)/(A+T);GC⁢skew=(G-C)/(G+C)

### Phylogenetic Analysis Based on Amino Acid Sequences of Mitochondrial Protein-Coding Genes

Amino acid translation of the PCGs in the mitochondrial genomes of *M. fructicola* and *M. laxa* isolates were obtained based on the mitochondrial translation code data four using the Geneious 9.1.8 program (Kearse et al., 2012). The phylogenetic tree was constructed using a concatenated amino acid matrix of the 14 core mitochondrial genes and ribosomal protein of *M. fructicola* and *M. laxa* isolates. To strengthen the evolutional relationships between our data and other genera of the Helotiales, amino acid sequences of each of the PCGs and ribosomal protein were included from published mitochondrial genome data. Additional datasets were obtained from NCBI GenBank under the following accession numbers; KC832409.1 (*Botrytinia fuckeliana*), KJ434027.1 (*Sclerotinia borealis*), KT283062.1 (*Sclerotinia sclerotiorum*), KF169905.1 (*Glarea lozoyensis*), NC_015789.1 (*Phialocephala subalpine*), KF650572.1 (*Rhynchosporium agropyri*), KF650575.1 (*Rhynchosporium secalis*), KF650573.1 (*Rhynchosporium commune*), and KF650574.1 (*Rhynchosporium orthosporum*). The multiple protein sequences were concatenated by using the Geneious 9.1.8 program (Kearse et al., 2012) and aligned by ClustalW using MEGA software version 7 (Kumar et al., 2016). The maximum likelihood (ML) was constructed using RAxmlGUI v2.0 (Silvestro and Michalak, 2012) with 1,000 bootstrap replicates under BLOSUM62 substitutional matrix. The phylogenetic tree was visualized by FigTree v1. 4. program (Rambaut, 2012) and rooted at the midpoint.

## Results

### Sequence Features of Mitogenomes of *Monilinia* Species

Most of the mitogenomes were extracted as one contig. However, the mitogenomes of five isolates (Yolkenari-1, Yildirim-2–10th, Ti-B3-A3-2, BG-B1-A17, and SC-B2-A4) were represented by four contigs. These contigs were mapped by using the other completed mitogenomes as reference. Statistics of QUAST reports for the assembled mitogenomes of 16 isolates of *Monilinia* species are provided in Table 2. After mapping, the mitogenome sizes ranged from 158,607 to 167,838 bp for *M. fructicola* and from 178,351 to 179,780 bp for *M. laxa.* Intraspecific length variations within isolates of *M. fructicola* were larger than the variations observed for the *M. laxa* isolates. An isolate of *M. laxa* (isolate code is MM-B2-A2) had the largest mitogenome size with 179,780 bp, while the isolate (coded as T-B1-A5) from *M. fructicola* species had the smallest mitogenome size with 158,607 bp. Total GC% content of all mitogenomes ranged between 30.0 and 31.1%.

**TABLE 2:** QUAST report for the 16 mitogenomes of *Monilinia* spp.

**Sample ID**	**Contigs**	**Largest contig**	**Total length**	**N50^∗^**	**N75**	**L50^∗∗^**	**L75**	**GC%**	**AT%**	**Species**
MM-B2-A2	1	179780	179780	179780	179780	1	1	30.09	69.91	*M. laxa*
2B1-A5	1	178431	178431	178431	178431	1	1	30.04	69.96	*M. laxa*
MT-B1-A3-1	1	178421	178421	178421	178421	1	1	30.05	69.95	*M. laxa*
Yildirim-1	1	178358	178358	178358	178358	1	1	30.06	69.94	*M. laxa*
Ni-B3-A2	1	178357	178357	178357	178357	1	1	30.06	69.94	*M. laxa*
MM-B4-A4	1	178353	178353	178353	178353	1	1	30.05	69.95	*M. laxa*
T-B1-A4-2	1	178351	178351	178351	178351	1	1	30.05	69.95	*M. laxa*
Ti-B3-A2	1	167471	167471	167471	167471	1	1	31.11	68.89	*M. fructicola*
B5-A4	1	159656	159656	159656	159656	1	1	30.95	69.05	*M. fructicola*
BG-B1-A4	1	159648	159648	159648	159648	1	1	30.94	69.06	*M. fructicola*
T-B1-A5	1	158607	158607	158607	158607	1	1	30.95	69.05	*M. fructicola*
Yolkenari-1	4	69540	149868	59243	59243	2	2	31.19	68.81	*M. fructicola*
Ti-B3-A3-2	4	66346	146595	59242	59242	2	2	31.21	68.79	*M. fructicola*
Yildirim-2-10^th^	4	64887	142596	56701	56701	2	2	31.05	68.95	*M. laxa*
BG-B1-A17	4	64885	142593	56701	56701	2	2	31.05	68.95	*M. fructicola*
SC-B2-A4	4	64884	142592	56701	56701	2	2	31.05	68.95	*M. fructicola*

*N50: length is at least half (50%) of the total base content of the assembly. **L50: is the number of contigs equal to or longer than the N50 length.

### Annotated Mitogenomes of Brown Rot Fungal Pathogens

**FIGURE 1:**
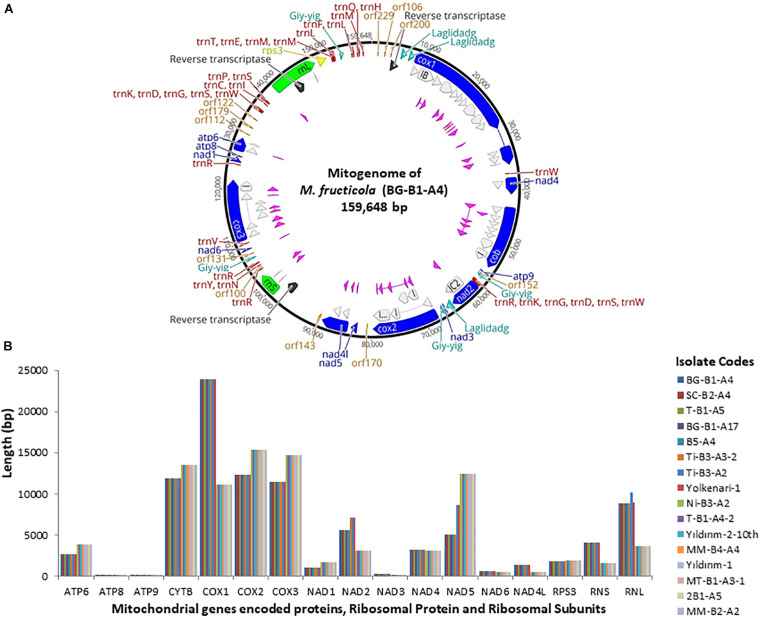
**(A)** Annotated reference circular mitogenomes of *M. fructicola*, showing core conserved protein-coding genes (blue) with introns-encoded homing endonucleases (pink and turquoise), reverse transcriptase (black), orf (orange), two ribosomal subunits (green), rps3 (yellow), introns (gray), and tRNAs (red). **(B)** Length variation of 14 protein-coding genes, two ribosomal subunits, and rps3 among 16 mitogenomes of *Monilinia* pathogens.

The annotated mitogenome, with circular structure, of *M. fructicola* (BG-B1-A4) was chosen as representative sequence and submitted for the first time to the NCBI GenBank (accession number MT005827) (Figure 1 and Supplementary Table 1). Besides, all the mitogenomes were submitted to NCBI GenBank and the accession numbers were provided in the “Data Availability Statement” section at the end of the paper. On the other hand, the complete mitogenome of the isolate Ni-B3-A2 of *M. laxa* species was submitted to the NCBI GenBank with accession number MN881998 by our previous study (Yildiz and Ozkilinc, 2020). Mitogenomes of the 16 isolates of *Monilinia* species had the 14 PCGs responsible for mitochondrial oxidative phosphorylation system (OXPHOS) and ATP synthesis, including cytochrome c oxidase subunits 1, 2, and 3 (*cox1-3*), NADH dehydrogenase subunits 1–6 and 4L (*nad1-6* and *nad4l*), ATP synthase subunits 6, 8, and 9 (*atp6-8-9*), and cytochrome b (*cob* or *cytb)*. Furthermore, two ribosomal RNA genes for large and small subunits (*rnl* and *rns*), 32 transfer RNA (*tRNAs*), and a gene-encoding ribosomal protein S3 (*rps3*) were annotated. A set of 32 tRNAs of *Monilinia* isolates encoded for 20 essential amino acids were involved in the mitochondrial protein synthesis (Figure 1). However, alanine (Ala) and cysteine (Cys) amino acids were absent in the mitochondrial tRNAs of *M. fructicola* and *M. laxa*, respectively. Moreover, for both species, some of the tRNA genes encoding different anticodons corresponded to the same amino acids. For instance, four copies of arginine amino acid, which were encoded by TCT and TCG anticodons (Supplementary Table 2). The AT-rich content for tRNA codons was observed in both species (Supplementary Table 2). The mitogenomes of *M. fructicola* and *M. laxa* also contained some unidentified open reading frames, represented as ORFs encoding hypothetical proteins, and those ORFs were conserved within the species.

The largest gene sizes among the mitochondrial PCGs in the isolates of *M. fructicola* were detected in *cytochrome c oxidase* (*cox*) subunits *1,2,3* and *cytochrome b* (*cytb*), ranging from 23.8 to 11 kb (Figure 1). The large-sized PCGs of *M. laxa* isolates were *cox1,2,3*, *cytb*, and *nad5* genes, ranging from 15.3 to 11 kb. Gene lengths were similar within the species but varied between the species. However, *atp8* and *atp9* genes showed the same size for all the 16, regardless of the species (Figure 1 and Supplementary Table 1). An unknown sequence with 1,219-bp length was detected within the *rnl* gene, only in the mitogenomes of the isolates of *M. fructicola*. This unknown sequence was not matched with any sequence in the NCBI gene bank. Furthermore, another duplication of this unknown sequence was detected within the *rnl* of the isolate named Ti-B3-A2, which has one of the largest mitogenome among all. On the other hand, these sequences were not present in any of the isolates of *M. laxa*.

### Skewness and Codon Usage Analysis

**FIGURE 2:**
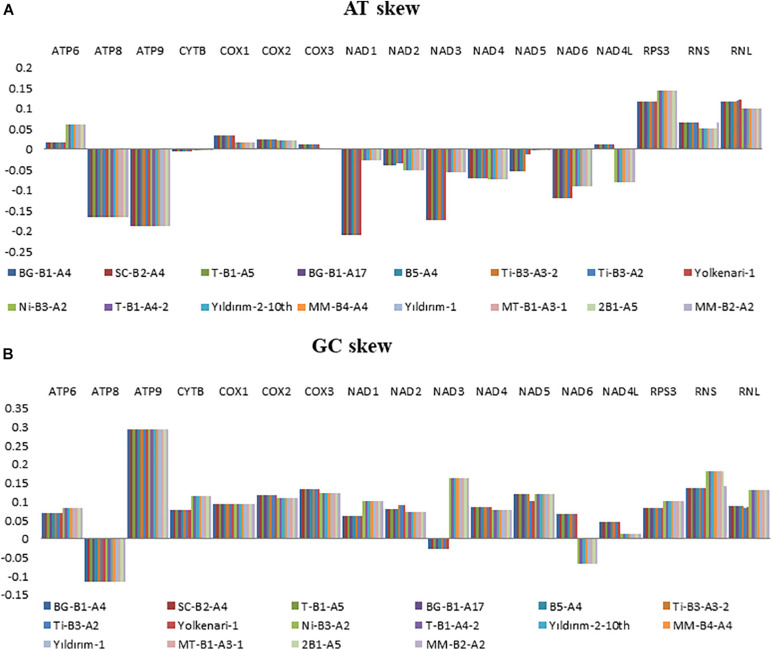
Graphical illustration showing the **(A)** AT- and **(B)** GC-skew in the mitochondrial genes of the 16 isolates of *Monilinia* spp.

Nucleotide contents of the 16 mitogenomes were represented according to their AT and GC skew values (Figure 2). Many of the PCGs showed negative AT skews for both species, except for the genes of *atp6*, *cox1*, *cox2*, and *cox3*, which exhibited a positive AT skew (Figure 2). The AT skew of *nad4l* varied among species as positive and negative asymmetry for *M. fructicola* and *M. laxa*, respectively. The GC skews of the core PCGs for 16 mitogenomes showed positive asymmetry except for *atp8* and *nad3*, which exhibited negative GC skews. Moreover, the GC skew of *nad6* was positive for the isolates of *M. fructicola* but negative for the isolates of *M. laxa*. In addition, genes encoding ribosomal subunits (*rnl*, *rns)* and ribosomal protein (*rps3*) showed positive AT and GC skewness in all the mitogenomes of *Monilinia* samples (Figure 2).

Codon usage analysis for the 14 mitochondrial PCGs and *rps3* indicated that the most frequently used codons are as shown in the Supplementary Table 3. Codon usage patterns were quite similar between the 16 mitogenomes. The total number of codons was the highest for Leucine (Leu), Isoleucine (Ile), Lysine (Lys), and Phenylalanine (Phe) amino acids in mitogenomes of *Monilinia* species (Supplementary Table 3).

### Comparison of Gene Arrangements of the Annotated Mitogenomes

**FIGURE 3:**
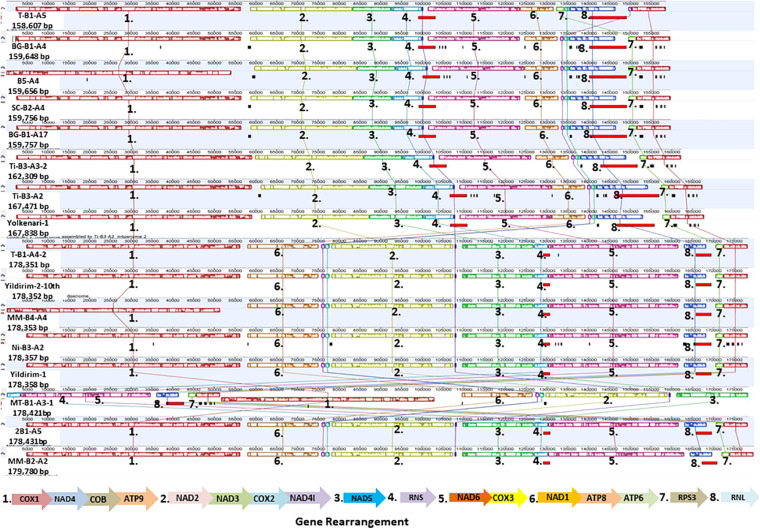
Gene order of 16 mitogenomes of *Monilinia* isolates is shown. Homologous regions are colored differently and numbered to follow the order.

The Mauve alignment reflected a conserved synteny among the 16 mitogenomes, which were divided into 11 homologous regions and represented with different colored synteny blocks (Figure 3). Gene order in the mitogenomes of *M. fructicola* isolates 3 was conserved with the exception for the order of the *rps3* gene, which differed in the isolate coded T-B1-A5 (Figure 3). *Rps3* was found as a free-standing gene in the mitogenomes analyzed. Gene order in the mitogenomes of *M. laxa* isolates was conserved within the species (Figure 3).

### Repetitive Sequences in the Mitogenomes of the Isolates of *Monilinia* species

Different repetitions were detected within the two species (Table 3). Isolates of *M. fructicola* presented two sequence motifs, which were (TAC)_29_ and (TC)_18_ located in the intergenic regions (Table 3). Among the eight mitogenomes of *M. fructicola*, 33–37 repeats were detected, and these repetitions covered 1.23–1.41% of the total mitogenome sizes. All the mitogenomes of *M. laxa* represented repetitive sequences in 59–60 bp in length comprising 1.60–1.69% of the total mitogenomes. The most longest repeats of more than 10 bp were (AT)_17_ in the mitogenome of *M. laxa* was detected previously (Yildiz and Ozkilinc, 2020). This repetition was found in the seven other isolates of the species. (AT)_17_ sequences were within an intron of the *cytb* gene, as reported previously (Yildiz and Ozkilinc, 2020).

**TABLE 3:** Information on repetitive motifs detected in the mitogenomes of 16 isolates of number *Monilinia* spp.

**Species**	**Isolate code**	Repeat motif*	Copy number of the repeat**	**Location of the repetition**	Total repeats***
*Monilinia laxa*	2B1-A5	AT	17	Intron of cob gene	60
	MM-B2-A2	AT	17	Intron of cob gene	60
	T-B1-A4-2	AT	17	Intron of cob gene	60
	Yildirim-1	AT	17	Intron of cob gene	60
	Yildirim-2-10^th^	AT	17	Intron of cob gene	60
	Ni-B3-A2	AT	17	Intron of cob gene	60
	MM-B4-A4	AT	17	Intron of cob gene	59
	MT-B1-A3-1	AT	17	Intron of cob gene	59
*Monilinia fructicola*	B5-A4	TAC	29	Intergenic region	37
		TC	18		
	BG-B1-A17	TAC	29	Intergenic region	36
		TC	18		
	SC-B2-A4	TAC	29	Intergenic region	36
		TC	18		
	T-B1-A5	TAC	29	Intergenic region	36
		TC	18		
	Ti-B3-A2	TAC	29	Intergenic region	36
		TC	18		
	BG-B2-A4	TAC	29	Intergenic region	36
		TC	18		
	Yolkenari-1	TAC	29	Intergenic region	34
		TC	18		
	Ti-B3-A3-2	TAC	29	Intergenic region	33
		TC	18		

*The repeat motif having the highest copy number among the all repeats found in the mitogenome is shown. **Copy number of the whole repeat units is given. ***All repeat units found in the mitogenome.

### Introns in the Mitogenomes of *Monilinia* Pathogens

**FIGURE 4:**
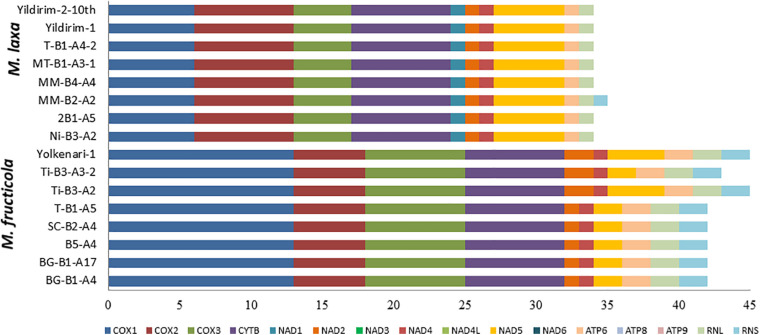
Intron size variations among 16 isolates of *Monilinia* spp.

Introns distributing within genic and intergenic regions, as well as mobile intron groups, were detected in all mitogenomes obtained from different isolates of two pathogenic species. The intron content of different genes varied in both species. In *M. fructicola*, the number of introns for each gene was *Cox1* with 13 introns, *cox2* with five introns, *cox3* with seven introns, *cytb* with seven introns, *nad2* and *nad4* with one intron, nad5 with two introns, atp6 with two introns, and large and small ribosomal subunit with four introns. On the other hand, *nad1*, *nad3*, *nad4l*, *atp8*, and *atp9* were found as intronless genes. Besides, some of the mitogenomes showed intron expansions within *nad2* and *nad5*. The isolates Ti-B3-A2, Ti-B3-A3-2, and Yolkenari-1 presented an additional intron (total 1,459 bp in size) in the *nad2* gene in comparison to the other samples. The *nad5* gene in the mitogenomes of the two isolates (Ti-B3-A2 and Yolkenari-1) included two extra introns (total 3,568 bp in size) to compare other isolates of the same species (Figure 4).

For all the eight mitogenomes of *M. laxa*, a total of 34 intron locations were found in *cox1* gene with six introns, *cox2* gene with seven introns, *cox3* gene with four introns, *cytb* gene with seven introns, *nad5* gene with five introns, *nad1* gene with one intron, as well as the *nad2*, *nad4*, *atp6*, and *rnl* genes with one intron. On the other hand, *nad3*, *nad4l*, *atp8*, *atp9*, and *rns* did not contain any intron. Furthermore, MM-B2-A4 had one extra intron within the *rns* gene encoding a small ribosomal subunit. The total number of introns were greater in the mitogenomes of *M. fructicola* than in the mitogenomes of *M. laxa*; however, the total intron lengths were larger in the mitogenomes of *M. laxa* (Figure 4). Introns in the mitogenomes of *M. fructicola* isolates covered 42.2% of the whole mitogenome, and within the non-coding regions (28%) and within coding regions (14.2%). The introns in mitogenomes of *M. laxa* isolates covered 38.9% of the whole mitogenome and were within the non-coding region (16.3%), and within coding sequences (22.6%).

For *M. fructicola* isolates, the most intronic sequence carrying gene was the *cytb* with 72.8%, and intron rich genes followed by *cox1* (66.5%), *cox2* (61.5%), and *cox3* (53%). The most intronic content was found in the *cox2* gene with 65.4% and followed by *nad1* (65.2%), *cytb* (62.1%), and *cox3* (53%) for *M. laxa* isolates. Besides, group I and group II mobile introns were detected in the mitogenomes of *M. fructicola* (Figure 1). Different LAGDIDADG and GIY-YIG elements encoding homing endonucleases were detected within genic and intergenic regions of the mitogenomes of *M. fructicola*. Each of these elements from group I was represented as a single copy. Representation of group I mobile introns was given in detail for one isolate of *M. laxa* in our previous study (Yildiz and Ozkilinc, 2020). Group I mobile introns encoding homing endonucleases were found as approximately 18.1% of the whole mitogenome of *M. fructicola* and 35.4% of the whole mt-genome of *M. laxa*. Moreover, three different sequences were annotated as group II introns in the mitogenomes of *M. fructicola*. These sequences were the same and located in the same positions within all mitogenomes of the isolates of *M. fructicola*. These sequences were annotated as encoding reverse transcriptase/maturase (Figure 1). Group II introns were not detected within the mitogenomes of *M. laxa*.

### Pan-Mitogenomics

**FIGURE 5:**
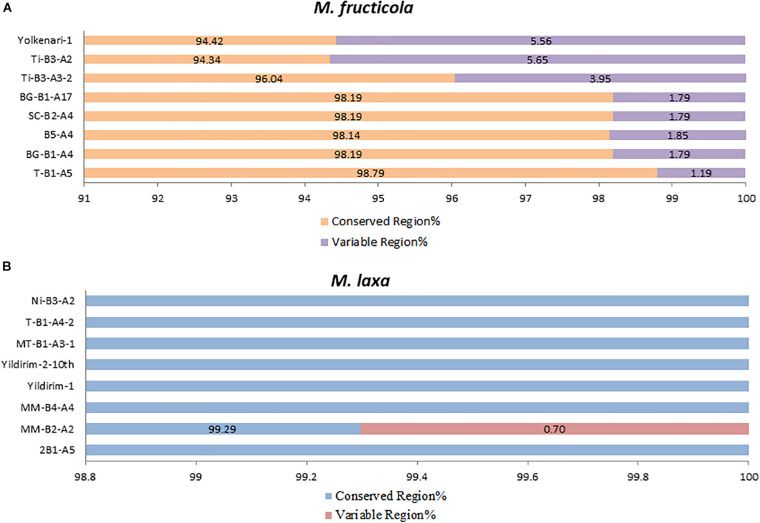
Predicted percentage of conserved and variable regions of the mitogenomes within each species **(A)** *M. fructicola* and **(B)** *M. laxa*.

The core mitochondrial genes included 14 PCGs, *rns*, *rnl*, and *rps3*. Except for *rnl*, *rns*, *nad2*, and *nad5*, all core genes were found fully conserved within species. Genic and intergenic introns, mobile introns (group I and group II), and repetitive sequences considered accessory regions of mitogenomes. However, many of these accessory elements were conserved within the species. The conserved regions formed a large portion of the mitogenome (ranging between 94 and 98%), while the variable regions covered 1.19–5.6% of the whole mitogenomes of the *M. fructicola* isolates. The most accessory-rich mitogenomes were detected in the isolates coded Ti-B3-A2 and Yolkenari-1 within *M. fructicola* (Figure 5). The whole mitogenomes of the isolates of *M. laxa* conserved within the species with the exception that one isolate (called MM-B4-A2) carried 0.7% of the total genome as a variable, which was not shared with any other isolate within this species (Figure 5).

According to the linear model test, intragenic introns had a significant effect (*P* < 0.0001 for both species) on mitogenome length variation. R^2^ values explaining dependent (mitogenome length) and independent (intron length) variables were found as 0.9851 and 0.995 for *M. fructicola* and *M. laxa*, respectively.

### Evolutionary Selection on Mitochondrial Genes of *Monilinia* Species

**FIGURE 6:**
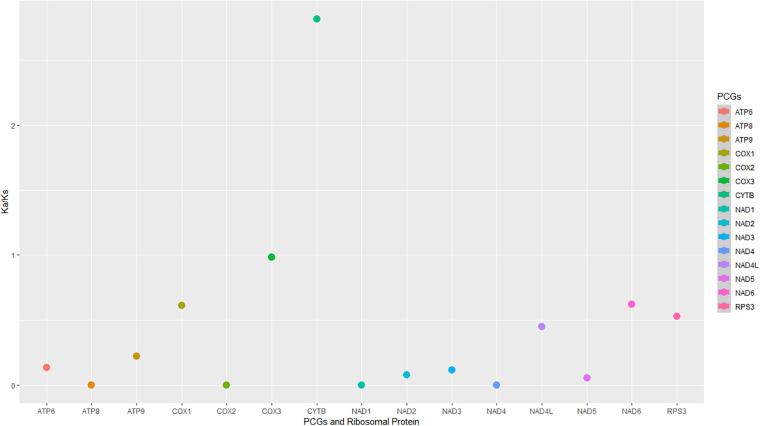
The non-synonymous/synonymous mutation rates (Ka/Ks) were of the protein-coding regions and rps3 in the 16 mitogenomes from *M. fructicola* and *M. laxa* species.

The evolutionary rates among the 16 mitogenomes from the two species showed that most of the genes were under negative (purifying selection) (Figure 6). The *Cox3* gene indicated neutral selection (Figure 6). The *cytb* gene was under diversifying or positive selection, and the remaining genes were under purifying selection (Figure 6).

### Maximum Likelihood Analysis of Mitochondrial Protein-Coding Genes of *Monilinia* spp. and Other Genera From the Heliotales

**FIGURE 7:**
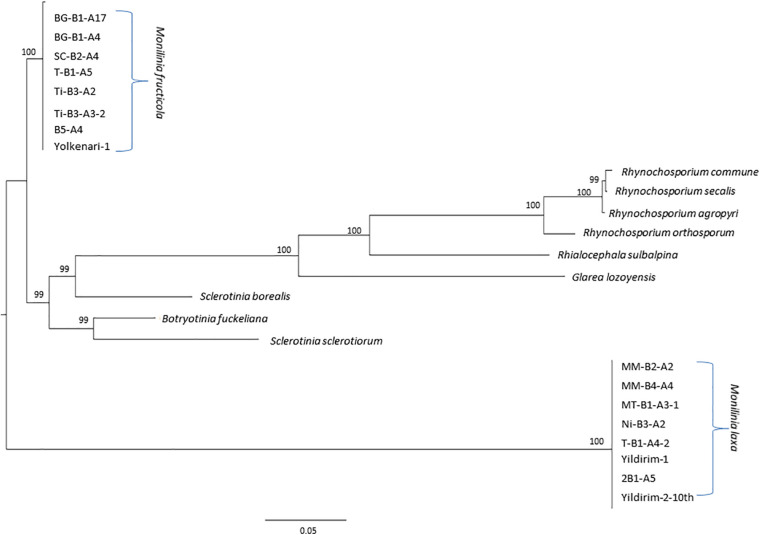
Maximum likelihood tree inferred from the dataset of amino acid sequences of the 14 core mitochondrial genes and ribosomal protein for *Monilinia* isolates used in this study and other species of the Helotiales obtained from the NCBI GenBank. Node numbers show bootstrap support values. Concatenated order follows as *cox1*, *nad4*, *cob (cytb)*, *atp9*, *nad2*, *nad3*, *cox2*, *nad4l*, *nad5*, *nad6*, *cox3*, *nad1*, *atp8*, *atp6*, and *rps3*. Node numbers show bootstrap support values.

The ML tree was obtained for the amino acid sequences of 14 PCGs and *rps3* of the *M. fructicola*, *M. laxa*, as well as some other species from Heliotales (Figure 7). Since all the amino acid sequences were conserved within the species, isolates were clustered together for each *Monilinia* species. However, *M. fructicola* and *M. laxa* diverged from each other with the high bootstrap value. Both *Monilinia* species were also distinctly related with the other species from Heliotales (Figure 7).

## Discussion

In this study, the complete mitogenomes of *M. fructicola* and *M. laxa* isolates were evaluated in-depth to understand variations within and between the species utilizing the pan-mitogenomic approach. The sizes of the 16 mitogenomes varied from 158,607 to 179,780 bp. The length of fungal mitogenomes is highly variable among the fungal species. It can range from 30 kb for the yeast *Candida parapsilosis* (Nosek et al., 2004) to 235 kb for *Rhizoctonia solani* (Losada et al., 2014). Based on the published data, mitogenomes of *Monilinia* pathogens seem to be quite large compared with other fungal mitogenomes. *S. borealis*, *S. sclerotiorum*, and *Botrytis cinerea*, closely related genera to *Monilinia*, presented mitogenomes of be 203, 128, and 82 kb in size, respectively (Mardanov et al., 2014). Mitogenome size differences are mainly related to the number and size of introns and repetitive elements that constitute the accessory part of the mitogenomes. Moreover, a variable number of genes of tRNAs, as well as loss/gain of some genes, such as *nad* and *atp* subunits, affect the mitogenome sizes (Aguileta et al., 2014; Franco et al., 2017; Li X. et al., 2020; Yildiz and Ozkilinc, 2020). In our study, the main factors that determined the differences in size of mitogenomes were introns, mobile intron groups, and repetitive sequences. Moreover, an unknown sequence and its duplicate was detected within the *rnl* gene within the isolates of *M. fructicola* and were not found in the mitogenomes of isolates of *M. laxa*. The origin and/or the duplication of this unknown sequence could be due to the mobility of mobile introns. Since this sequence was found within an rRNA-encoding gene, a group I intron-encoding RNA working as a ribozyme could be the source for this sequence. This further indicates that dynamism has been shaping the evolution and structure of the fungal mitogenomes continuously.

Fungal mitogenomes are known to have a high AT content, as confirmed in this study as well as in previous studies (Torriani et al., 2014; Franco et al., 2017). The high GC content of genomes was reported to affect the genome to evolve the advantage to maintain DNA stability in the high temperature, UV exposure, and fungicides (Musto et al., 2006; Marsolier-Kergoat, 2013). Furthermore, GC content has an important effect on evolutionary selection, recombination, gene conversion, and recombination in fungal plant pathogens (Stukenbrock and Dutheil, 2018). These considerations can be extended to mitogenomes as well.

Fungal mitogenomes have clustered with many tRNA genes with the different anticodons, indicating a strong preference for A or T, in the third position of codons. This strong preference using A/T has been defined in other species as wobble pairing and codon usage bias (Novoa and de Pouplana, 2012; Wei et al., 2014). However, having the decoders or iso-acceptors may cause mischarging (Pan, 2018), but this situation was not discussed or shown in any fungal mitochondrial genome.

Repetitive elements were 1.23–1.69% in the mitogenomes assessed in this study. The total number of these elements was found greater and more diverse in *M. laxa* than in *M. fructicola*. Expansion of repetitive elements may have caused replication slippage and the correction of mitochondrial replication process together with proofreading efficiency may differ among the species. It is known that repeat-rich areas evolve more rapidly than other genomic regions (Raffaele and Kamoun, 2012; Dong et al., 2015). If the repeat-rich regions locate within genic regions, changes in these elements may indicate evolutionary selections of certain traits such as resistance or host adaptation (Raffaele et al., 2010; Nardi et al., 2012). Furthermore, exploring repetitive elements in mitogenomes could be highly useful in population genetics analyses, and they could have an important role for the dynamic structure of mitogenomes. Besides, mitochondrial repetitive elements can be useful molecular markers to study population structures.

Core PCGs related to mitochondrial OXPHOS and ATP synthesis are usually essential for the organisms’ life and, thus, highly conserved within the mitogenomes. However, accessory regions could affect some traits, such as pathogenesis and virulence reference (Tettelin et al., 2008), and not be crucial for the survival of the organism. We observed a negative correlation between mitogenome size and virulence degree for these isolates (unpublished data by HÖ). It is well known that accessory regions in the nuclear genomes may change to adapt to evolutionary processes among the fungal isolates of the same species (Plissonneau et al., 2018; Badet and Croll, 2020). However, the effects of accessory regions of mitogenomes on different fungal traits have not been clarified. Mobile introns are another primary source of the size difference for mitogenomes that are highly found in both *Monilinia* species. Our previous study showed that *M. laxa* was the most intron-rich species compared with closely related species from other genera (Yildiz and Ozkilinc, 2020). These mobile introns included many different elements from group I in both species as well as group II introns in *M. fructicola*. Relatedness and phylogenetic signals of these elements within and between *Monilinia* species is another question that requires further investigations. Besides, these elements may have a contribution to certain traits such as virulence or fungicide resistance, and our ongoing studies have been directed to answer those questions.

Pan-mitogenomics approach identified that the core and accessory compartmentalizations occurred within *M. fructicola* species. Even though isolates of *M. laxa* carried many different introns, mobile elements, and repetitive sequences considered as an accessory part of the genomes, these sequences were mainly conserved among the mitogenomes within this species. Only one isolate of *M. laxa* diverged from the other *M. laxa* isolates with a unique region that was represented as a variable part for the mitogenomes of *M. laxa*. This approach indicates that introns, mobile groups, and repetition patterns are highly conserved and stable within the mitogenomes of *M. laxa*. In contrast, the mitogenomes of *M. fructicola* showed variability and dynamism within the species. This could be related to possible recombination and/or selection pressures on mitogenomes of *M. fructicola*.

A/T and G/C skewness varied among the PCGs as well as between the species for some genes. Interestingly, genes related to ribosomal RNA and protein synthesis were in positive G/C and A/T skewness, indicating the richness of G and A over C and T, respectively. GC compositions were also interpreted as related to transcription start sites in plants and fungi (Fujimori et al., 2005). The different skewness along the regions may also indicate diverse selection pressures on two species. This study indicated that most of the coding genes have been evolving under strong purifying selection between the species according to the Ka/Ks ratios. Diversely, *cytb* was under a positive selection signal between the species. *Cytb* gene is one of the target regions of respiratory inhibitors that have been intensively used against fungal plant pathogens. Positive selection signals on this gene could be related to different adaptation responses of the species against fungicide selection pressure, which will be discussed in our further study for these pathogens.

Amino acid sequences of the core mitochondrial PCGs and *rps3* were fully conserved within *M. fructicola* and *M. laxa* species. Combined protein-coding data set based on ML phylogenetic indicated that these two species are highly diverged from each other as well as some from other fungal species from the Heliotales group. However, since one of the main variation contributors is the mobile introns, phylogenetic effects of these elements would be interesting. Mobile introns may shape evolutionary relationships differently in comparison with core PCGs as presented by Megarioti and Kouvelis (2020). Besides, mobile introns may reveal evolving lineages within each fungal species, and this will be investigated in a further study.

Considerable mitogenomic variations were observed within and between these two important pathogenic species within the *Monilinia* genus. Fungal mitochondrial genomes are still waiting for many hidden information on fungal traits and evolution. Pangenomic approach is successfully applicable for fungal mitogenomes due to relatively expanded accessory regions, as shown in this study. Since these organisms are important plant pathogens worldwide, resolving their mitogenomes may suggest new disease management strategies and predictions in evolutionary trajectories of the pathogens and the disease.

## Data Availability Statement

The first mitogenome of *M. fructicola* was submitted in this study can be found in GenBank (Accession number MT005827). Besides, the mitogenome of *M. laxa* used in this study, was submitted in our previous research and can be found in GenBank (Accession number MN881998.1). All the mitogenomes was submitted to GenBank with following accession numbers for each isolate used in this study: MW794295 for isolate B5-A4, MW794296 for isolate BG-B1-A17, MW794297 for isolate SC-B2-A4, MW794298 for T-B1-A5, MW794299 for isolate Ti-B3-A2, MW794300 for isolate Ti-B3-A3-2, MW794301 for isolate Yolkenari-1, MW794302 for isolate Yildirim-2-10th, MW794303 for isolate Yildirim-1, MW794304 for isolate T-B1-A4-2, MW794305 for isolate MT-B1-A3-1, MW794306 for isolate MM-B4-A4, MW794307 for isolate MM-B2-A2, and MW794308 for isolate 2B1-A5.

## Author Contributions

GY performed laboratory work and all the data analyses. HO designed the research, recommended, and directed the data analysis methods, and controlled all steps of the study. Both authors confirmed and discussed the results and wrote the article together.

## Conflict of Interest

The authors declare that the research was conducted in the absence of any commercial or financial relationships that could be construed as a potential conflict of interest.

## Footnotes

1 https://www.ncbi.nlm.nih.gov/genome

2 https://www.ncbi.nlm.nih.gov/genome/organelle/

## Supplementary Material

Supplementary Table 1 Characterization of annotated mitochondrial genes in *Monilinia fructicola-*Isolate BG-B1-A4, accession number: MT005827.

Supplementary Table 2 Set of 32 tRNAs encoding different anticodons in the mitogenomes of *Monilinia fructicola* and *Monilinia laxa* species.

Supplementary Table 3 Dataset of the relative synonymous codon usage (RSCU) and the total number of usages for each of the codons in the 14 mitochondrial PCGs, and rps3.
