# Supplementary material for: Pan-Mitogenomics Approach Discovers Diversity and Dynamism in the Prominent Brown Rot Fungal Pathogens
Source: Front Microbiol. 2021 May 12;12:647989. doi: 10.3389/fmicb.2021.647989 (PMC8149612; doi:10.3389/fmicb.2021.647989)
Supplement: Supplementary Table 1 — Characterization of annotated mitochondrial genes in Monilinia fructicola-Isolate BG-B1-A4, accession number: MT005827. [file Table_1.docx]

**Supplementary Table 1** Characterization of annotated mitochondrial genes in *Monilinia fructicola-*Isolate BG-B1-A4, accession number: MT005827

| **Gene** | **Start**  **Position** | **Stop**  **Position** | **Length**  **(nt)** | **Start Codon** | **Stop Codon** | **GC % Contents** | **Product** |
| --- | --- | --- | --- | --- | --- | --- | --- |
| ORF229 | 1.201 | 1.356 | 156 | ATG | TAA | 41.0 | Hypothetical protein |
| ORF106 | 1.781 | 2.395 | 615 | ATG | TAA | 34.6 | Hypothetical protein |
| ORF200 | 2.798 | 2.908 | 111 | ATG | TAA | 43.2 | Hypothetical protein |
| Cox1 | 8.142 | 28.525 | 20.384 | ATG | TAA | 30.0 | Cytochrome c oxidase subunit 1 |
| Cox1-trun | 31.984 | 35.479 | 3.497 | ATG | TAA | 30.0 | Truncated cox1 |
| trnW | 37.022 | 37.092 | 71 | - | - | 35.2 | Transfer RNA Tryptophan |
| Nad4 | 37.745 | 40.943 | 3.199 | ATG | TAA | 30.4 | NADH dehydrogenase subunit 4 |
| Cytb/Cob | 43.173 | 55.099 | 11.927 | ATG | TAA | 30.8 | Cytochrome b |
| Atp9 | 56.130 | 56.354 | 225 | ATG | TAA | 36.4 | ATP synthase F0 subunit c |
| ORF152 | 56.440 | 56.844 | 405 | TTA | TAG | 32.3 | Hypothetical protein |
| trnR | 58.091 | 58.161 | 71 | - | - | 33.8 | Transfer RNA Arginine |
| trnK | 58.239 | 58.309 | 71 | - | - | 31.0 | Transfer RNA Arginine |
| trnG | 58.354 | 58.424 | 71 | - | - | 47.9 | Transfer RNA Glycine |
| trnD | 58.426 | 58.498 | 73 | - | - | 41.1 | Transfer RNA Aspartic acid |
| trnS | 58.530 | 58.609 | 80 | - | - | 35.0 | Transfer RNA Serine |
| trnW | 58.654 | 58.724 | 71 | - | - | 35.2 | Transfer RNA Tryptophan |
| Nad2 | 58.725 | 64.372 | 5.648 | ATG | TAA | 31.6 | NADH dehydrogenase subunit 2 |
| Nad3 | 65.847 | 66.173 | 327 | TTG | TAA | 33.3 | NADH dehydrogenase subunit 3 |
| Cox2 | 67.665 | 79.941 | 12.277 | ATG | TAG | 30.4 | Cytochrome c oxidase subunit 2 |
| ORF170 | 80.895 | 81.056 | 162 | ATG | TAA | 27.8 | Hypothetical protein |
| Nad4l | 82.669 | 84.015 | 1.347 | ATG | TAG | 28.7 | NADH dehydrogenase subunit 4l |
| Nad5 | 84.444 | 89.463 | 5.020 | ATG | TAA | 31.8 | NADH dehydrogenase subunit 5 |
| ORF143 | 89.933 | 90.376 | 444 | ATG | TAA | 36.9 | Hypothetical protein |
| rns | 99.091 | 103.121 | 4.031 | - | - | 33.8 | Small subunit ribosomal RNA |
| trnR | 104.127 | 104.198 | 72 | - | - | 48.6 | Transfer RNA Arginine |
| ORF100 | 104.376 | 104.678 | 303 | ATG | TAA | 36.0 | Hypothetical protein |
| trnY | 104.834 | 104.918 | 85 | - | - | 34.1 | Transfer RNA Tyrosine |
| trnN | 105.485 | 105.555 | 71 | - | - | 39.1 | Transfer RNA Asparagine |
| trnR | 105.577 | 105.648 | 72 | - | - | 48.6 | Transfer RNA Arginine |
| ORF131 | 107.576 | 107.725 | 150 | ATG | TAA | 26.0 | Hypothetical protein |
| Nad6 | 108.362 | 108.958 | 597 | ATG | TAG | 23.8 | NADH dehydrogenase subunit 6 |
| trnV | 109.701 | 109.772 | 72 | - | - | 44.4 | Transfer RNA Valine |
| Cox3 | 110.349 | 121.856 | 11.508 | ATG | TAG | 31.0 | Cytochrome c oxidase subunit 3 |
| trnR | 124.661 | 124.731 | 71 | - | - | 33.8 | Transfer RNA Arginine |
| Nad1 | 124.968 | 126.059 | 1.092 | ATG | TAG | 32.4 | NADH dehydrogenase subunit 1 |
| Atp8 | 126.199 | 126.345 | 147 | ATG | TAA | 23.1 | ATP synthase F0 subunit b |
| Atp6 | 127.042 | 129.713 | 2.672 | ATG | TAA | 28.8 | ATP synthase F0 subunit a |
| ORF112 | 130.559 | 130.721 | 163 | ATT | TAG | 36.2 | Hypothetical protein |
| ORF179 | 131.825 | 132.364 | 540 | ATG | TAG | 32.2 | Hypothetical protein |
| ORF122 | 133.572 | 133.940 | 369 | ATG | TAA | 40.7 | Hypothetical protein |
| trnK | 135.428 | 135.499 | 72 | - | - | 34.7 | Transfer RNA Lysine |
| trnG | 135.544 | 135.614 | 71 | - | - | 47.9 | Transfer RNA Glycine |
| trnD | 135.616 | 135.688 | 73 | - | - | 41.1 | Transfer RNA Aspartic acid |
| trnS | 135.720 | 135.799 | 80 | - | - | 35.0 | Transfer RNA Serine |
| trnW | 135.844 | 135.914 | 71 | - | - | 35.2 | Transfer RNA Tryptophan |
| trnC | 137.097 | 137.169 | 73 | - | - | 32.9 | Transfer RNA Cysteine |
| trnI | 137.486 | 137.557 | 72 | - | - | 38.9 | Transfer RNA Isoleucine |
| trnS | 137.660 | 137.745 | 86 | - | - | 38.4 | Transfer RNA Serine |
|  |  |  |  |  |  |  |  |
| trnP | 137.761 | 137.833 | 73 | - | - | 46.6 | Transfer RNA Proline |
| rnl | 140.233 | 149.116 | 8.884 | - | - | 34.1 | Large subunit ribosomal RNA |
| rps3 | 149.594 | 151.357 | 1.764 | - | - | 28.3 | Ribosomal Protein |
| trnT | 152.204 | 152.274 | 71 | - | - | 42.3 | Transfer RNA Threonine |
| trnE | 152.443 | 152.515 | 73 | - | - | 42.5 | Transfer RNA Valine |
| trnM | 152.544 | 152.614 | 71 | - | - | 39.4 | Transfer RNA Methionine |
| trnM | 152.655 | 152.727 | 73 | - | - | 43.8 | Transfer RNA Methionine |
| trnL | 152.895 | 152.977 | 83 | - | - | 44.6 | Transfer RNA Leucine |
| trnF | 156.085 | 156.157 | 73 | - | - | 45.2 | Transfer RNA Phenylalanine |
| trnL | 156.328 | 156.412 | 85 | - | - | 35.3 | Transfer RNA Leucine |
| trnQ | 157.138 | 157.211 | 74 | - | - | 45.9 | Transfer RNA Glutamine |
| trnH | 157.430 | 157.502 | 73 | - | - | 41.1 | Transfer RNA Histidine |
| trnM | 158.177 | 158.249 | 73 | - | - | 31.5 | Transfer RNA Methionine |
