# Supplementary material for: Pan-Mitogenomics Approach Discovers Diversity and Dynamism in the Prominent Brown Rot Fungal Pathogens
Source: Front Microbiol. 2021 May 12;12:647989. doi: 10.3389/fmicb.2021.647989 (PMC8149612; doi:10.3389/fmicb.2021.647989)
Supplement: Supplementary Table 2 — Set of 32 tRNAs encoding different anticodons in the mitogenomes of Monilinia fructicola and Monilinia laxa species. [file Table_2.docx]

**Supplementary Table 2** Set of 32 tRNAs encoding different anticodons in the mitogenomes of *Monilinia fructicola* and *Monilinia laxa* species

| ***Monilinia fructicola*** |  |  |  |
| --- | --- | --- | --- |
| **Amino acid** | **Anticodon** | **tRNA Symbol** | **Number** |
| Ala | - | trnA | 0 |
| Arg | TCT | trnR | 2 |
| Arg | TCG | trnR | 2 |
| Asn | GTT | trnN | 1 |
| Asp | GTC | trnD | 2 |
| Cys | GCA | trnC | 1 |
| Gln | TTG | trnQ | 1 |
| Glu | TTC | trnE | 1 |
| Gly | TCC | trnG | 2 |
| His | GTG | trnH | 1 |
| IIe | GAT | trnI | 1 |
| Leu | TAA | trnL | 1 |
| Leu | TAG | trnL | 1 |
| Lys | TTT | trnK | 2 |
| Met | CAT | trnM | 3 |
| Phe | GAA | trnF | 1 |
| Pro | TGG | trnP | 1 |
| Ser | GCT | trnS | 2 |
| Ser | TGA | trnS | 1 |
| Thr | TGT | trnT | 1 |
| Trp | TCA | trnW | 3 |
| Tyr | GTA | trnY | 1 |
| Val  ***Monilinia laxa*** | TAC | trnV | 1 |
| **Amino acid** | **Anticodon** | **tRNA Symbol** | **Number** |
| Ala | TGC | trnA | 1 |
| Arg | TCT | trnR | 2 |
| Arg | TCG | trnR | 2 |
| Asn | GTT | trnN | 1 |
| Asp | GTC | trnD | 2 |
| Cys | - | trnC | 0 |
| Gln | TTG | trnQ | 1 |
| Glu | TTC | trnE | 1 |
| Gly | TCC | trnG | 2 |
| His | GTG | trnH | 1 |
| Ile | GAT | trnI | 1 |
| Leu | TAA | trnL | 1 |
| Leu | TAG | trnL | 1 |
| Lys | TTT | trnK | 2 |
| Met | CAT | trnM | 3 |
| Phe | GAA | trnF | 1 |
| Pro | TGG | trnP | 1 |
| Ser | GCT | trnS | 2 |
| Ser | TGA | trnS | 1 |
| Thr | TGT | trnT | 1 |
| Trp | TCA | trnW | 3 |
| Tyr | GTA | trnY | 1 |
| Val | TAC | trnV | 1 |
